# Hemodynamic effects of the carotid abnormalities courses by MRI and ultrasound

**DOI:** 10.1186/1532-429X-17-S1-P415

**Published:** 2015-02-03

**Authors:** Yuliya Stankevich, Mariya Rezakova, Bogomyakova Olga, Liubov Shraibman, Andrey Tulupov

**Affiliations:** 1 International Tomography Center SB RAS, Novosibirsk, Russian Federation; 2 Institute of Physiology and Fundamental Medicine SB RAMS, Novosibirsk, Russian Federation; 3 Novosibirsk State University, Novosibirsk, Russian Federation

## Background

The aim of our investigation was to determine effect of the internal carotid arteries (ICAs) tortuosity (S-shape), kinking and coiling on the arterial blood flow by magnetic resonance imaging and ultrasound (US).

## Methods

**Figure 1:**
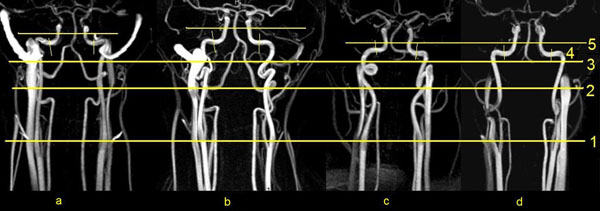
Investigated ICA segments in normal course (a) and abnormalities course (b - S-shape, c - coiling, d - kinking): 1 - C1, cervical segment (proximal part); 2 - C1, cervical segment (middle part); 3 - C2, petrous segment (vertical part); 4 - C2, petrous segment (horizontal part); 5 - C5, cavernous segment.

50 healthy volunteers (control group) and 43 patients with carotid abnormalities (CAs) were examined on 1.5T MR-scanner using routine MR protocol and quantitative MR angiography (qMRA) for estimate of the arterial blood flow velocity and cross sectional area of the ICA. We observed the cross sectional area and the values of peak velocity, mean velocity, flux velocity of arterial blood flow at one cardiac cycle at cervical and intracranial segments of the ICA (Fig. 1). 20 volunteers were performed US for the assessment of the vascular wall and peak systolic velocity (PSV) values of the arterial blood at the portion between common carotid artery (CCA) bifurcation and ICA. Statistical analysis was performed by mean value and confidence interval (for p=0.05). Student's paired t-test was used to indicate significant differences between mean values at the patient and control groups.

## Results

**Figure 2:**
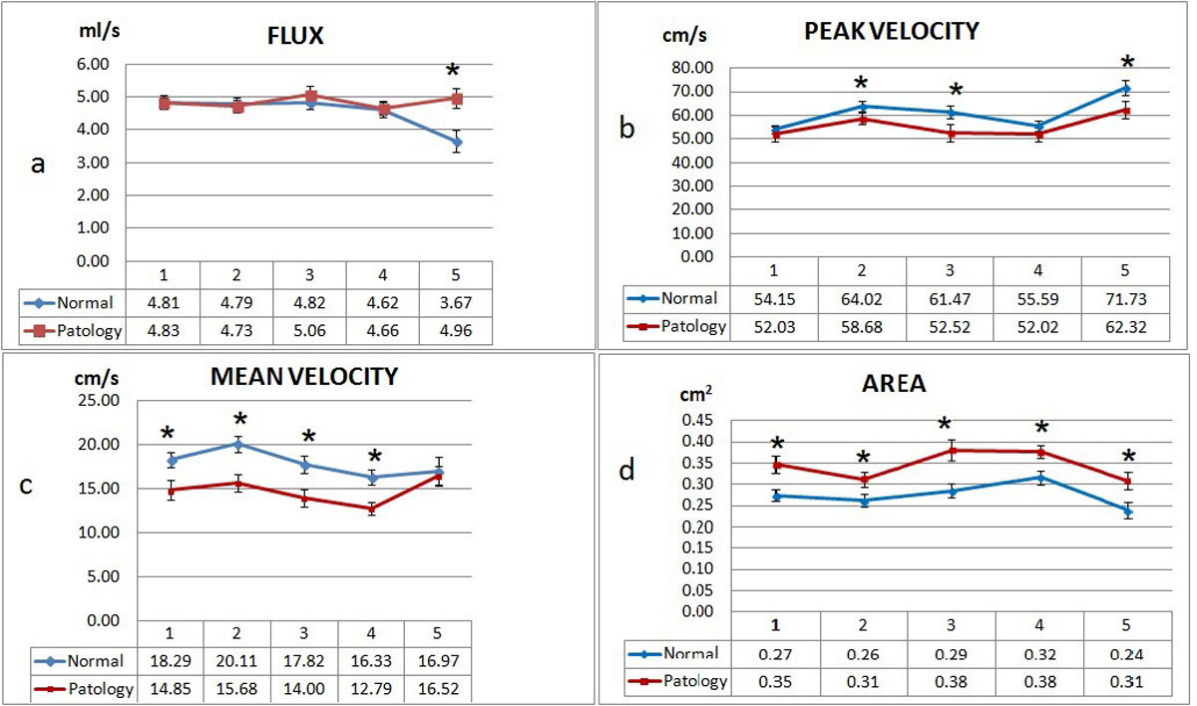
Cross-sectional area (d) and arterial blood flow velocity parameters (a - flux velocity, b - peak velocity, c - mean velocity) at different ICA segments. * - significant differences between values cross-sectional area and arterial blood flow velocity parameters of the ICA at control groups (n=100) and CAs group (n=67), p=0,05

The shapes of the graphs of the mean velocity depending on the phase of the cardiac cycle were the same in the case of the control and CAs groups obtained by MRI and US. The values of blood flow velocity parameters were significant differences at different segments of the ICA in the control and CAs groups (Fig. 2). There were no significant differences between values of the arterial blood velocity parameters in case of the tortuosity (S-shape), kinking and coiling at different segments of ICA. The cross sectional area of the vessel was significantly higher at the all investigated parts in case of CAs (Fig. 2). The correlation coefficient between the values of peak systolic velocity obtained by ultrasound and MRI was low (-0.01)

## Conclusions

Type of the artery pulsation at CAs is the same as the control group and corresponds to the musculo-elastic arteries. CAs affects the hemodynamic throughout the artery whith a background of increasing the area of its cross section. The ultrasound has confirmed the results obtained on MRI qualitatively, but correlation coefficient for quantitative parameters was low.

## Funding

This work was financially supported by the Russian Science Foundation (project # 14-35-00020).

